# Small extracellular vesicles derived from synovial fibroblasts contain distinct miRNA profiles and contribute to chondrocyte damage in osteoarthritis

**DOI:** 10.1186/s13075-024-03398-3

**Published:** 2024-09-28

**Authors:** Sabha Asghar, Gary J. Litherland, John J. Cole, Iain B. McInnes, R. M. D. Meek, John C. Lockhart, Carl S. Goodyear, Anne Crilly

**Affiliations:** 1 School of Health and Life Sciences, University of the West of Scotland, Hamilton International Technology Park, Lanarkshire Campus, Stephenson Place, South Lanarkshire, G72 0LH Scotland, UK; 2 School of Infection and Immunity, University of Glasgow, Glasgow, G12 8TA Scotland, UK; 3 Orthopaedic Department, Queen Elizabeth University Hospital (QEUH), Glasgow, Scotland, UK

**Keywords:** Osteoarthritis, Small extracellular vesicles, Synovial fibroblasts, Chondrocytes, microRNA, miR182

## Abstract

**Background:**

Small extracellular vesicles (sEV) derived from synovial fibroblasts (SF) represent a novel molecular mechanism regulating cartilage erosion in osteoarthritis (OA). However, a comprehensive evaluation using disease relevant cells has not been undertaken. The aim of this study was to isolate and characterise sEV from OA SF and to look at their ability to regulate OA chondrocyte effector responses relevant to disease. Profiling of micro (mi) RNA signatures in sEV and parental OA SF cells was performed.

**Methods:**

SF and chondrocytes were isolated from OA synovial membrane and cartilage respectively (*n* = 9). sEV were isolated from OA SF (± IL-1β) conditioned media by ultracentrifugation and characterised using scanning electron microscopy (SEM) and transmission electron microscopy (TEM). Particle size was confirmed by nanoparticle tracking analysis (NTA). sEV regulation of OA chondrocyte and cartilage effector response was evaluated using qPCR, ELISA and sulphated glycosaminoglycan assay (sGAG). RNA-sequencing was used to establish miRNA signatures in isolated sEV from OA SF.

**Results:**

OA SF derived sEV were readily taken up by OA chondrocytes, with increased expression of the catabolic gene *MMP 13* (*p* < 0.01) and decreased expression of the anabolic genes *aggrecan* and *COL2A1* (*p* < 0.01) observed. Treatment with sEV derived from IL-1β stimulated OA SF significantly decreased expression of *aggrecan* and *COL2A1* (*p* < 0.001) and increased *SOX 9 gene* expression (*p* < 0.05). OA chondrocytes cultured with sEV from either non-stimulated or IL-1β treated OA SF, resulted in a significant increase in the secretion of IL-6, IL-8 and MMP-3 (*p* < 0.01). Cartilage explants cultured with sEV from SF (± IL-1β) had a significant increase in the release of sGAG (*p* < 0.01). miRNA signatures differed between parental SF cells and isolated sEV. The recently identified osteoclastogenic regulator miR182, along with miR4472-2, miR1302-3, miR6720, miR6087 and miR4532 were enriched in sEV compared to parental cells, *p* < 0.01. Signatures were similar in sEVs derived from non-stimulated or IL-1β stimulated SF.

**Conclusions:**

OA SF sEV regulate chondrocyte inflammatory and remodelling responses. OA SF sEV have unique signatures compared to parental cells which do not alter with IL-1β stimulation. This study provides insight into a novel regulatory mechanism within the OA joint which could inform future targeted therapy.

**Supplementary Information:**

The online version contains supplementary material available at 10.1186/s13075-024-03398-3.

## Introduction

Osteoarthritis (OA) is the most prevalent of the musculoskeletal conditions and represents a significant public health burden. Compared with other inflammatory arthropathies, advancement in treatments for management of OA has been limited, with many patients suffering with years of pain and disability before being offered joint replacement. While degeneration of articular cartilage is a key feature of OA, it is now recognised as a complex condition affecting the whole joint, with synovial membrane inflammation present in around 70% of patients, and synovitis correlating with pain and cartilage damage [1–4]. In normal joint homeostasis, synovial fibroblasts (SF) are responsible for the secretion of synovial fluid which nourishes, lubricates and protects the joint. As an avascular, aneural and alymphatic tissue, this process is essential for maintenance of healthy cartilage. During OA it has been established that SF are a rich source of inflammatory mediators, including cytokines and, matrix metalloproteinases (MMP), which contribute to cartilage degeneration through activation of catabolic pathways and enhancement of matrix degrading enzymes [5, 6]. In addition to these inflammatory proteins, studies have described a role for micro (mi)RNAs in the destruction of OA cartilage, with many of these shown to modulate multiple anabolic/catabolic transcription and proteinase factors [7]. Consistent with these findings, a number of miRNA have been shown to be differentially regulated in OA cartilage and synovial membrane [8–12].

Small extracellular vesicles or sEV are a sub-type of extracellular vesicle, previously defined as exosomes, which range in size from 30 to 120 nm [13, 14]. They arise via the endocytic pathway, from the endosomal cell compartment, where they are stored in multivesicular bodies (MVBs) of late endosomes and released in short bursts by exocytosis upon fusion with the cell membrane [15–17]. sEV release occurs naturally from many cells and is recognised to play an important role in cellular communication via the transport of biological cargo [18]. Multiple cell types, including chondrocytes and SF from non-diseased joints have been shown to be capable of sEV secretion, with regulation of several mediators linked to OA pathogenesis reported [19–21].

sEV cargo includes mRNA transcripts and miRNA as well as small noncoding RNA species, repeat sequences, structural RNAs, tRNA fragments, vault RNA, Y RNA and small interfering RNAs, with some indication of active packaging depending on parental cell type [22, 23, 24]. Multiple studies have shown that transport of mRNA and protein occurs through exosomal machinery, with the genetic material functionally transferred with successful translation of proteins affecting cellular phenotype [18, 25–28]. Further studies have confirmed that this horizonal transfer of mRNA requires the presence of RNA in the recipient cell [29]. Identifying the miRNA content of sEV has been a focus of research since the majority of circulating miRNA is believed to be sequestered within this type of vesicle. These sEV offer protection from circulating RNAses, allowing miRNA to retain the ability to control gene expression by regulating target-mRNA turnover [28, 30]. Differences in specific RNA cargo has consistently been observed between healthy and disease states [24].

While sEV have been observed in OA synovial fluid [31–33], their cellular source and role in disease pathogenesis is unclear. Investigation of sEV source and cargo during OA could elucidate regulatory mechanisms within the joint, providing insight for targeted treatment. Based on current knowledge, our study hypothesised that sEV derived from OA SF carry biological cargo capable of regulating cartilage catabolic responses. The aim of this study was to isolate and characterise sEV originating from disease relevant OA SF and to look at their ability to regulate chondrocyte and cartilage effector responses. Using RNA sequencing (RNA-Seq), sEV miRNA signatures and that of their parental cells were also investigated.

## Methods

### Tissues and ethical approval

Matched synovial membrane and cartilage were collected from OA patients (*n* = 24 across the study) undergoing joint arthroplasty and tissue used to prepare primary cells. Ethics approval for the study was given by NHS Greater Glasgow and Clyde, (REC Ref # 10/S0704/60), with patient written informed consent obtained prior to tissue collection.

### Isolation of primary OA synovial fibroblasts (SF) and chondrocytes

Synovial membrane was harvested from joint arthroplasty before being digested with Liberase (Liberase TM, Roche UK; used at 5 mg/ml). Tissue was agitated at 37^o^C for 1.5–2 h to obtain a single cell suspension which was filtered through a 0.2 μm sterile filter (ThermoFisher Scientific, UK), centrifuged at 1200 rpm for 10 min and washed in phosphate buffered saline (PBS) before resuspension. The isolated cells were transferred to T75 vented flasks and cultured in complete Dulbecco’s Modified Eagle’s Medium or DMEM (with Glutamax and 2% penicillin streptomycin solution) supplemented with 10% foetal calf serum (FCS; Sigma UK), at 37^o^C/ 5% CO_2_. Experiments were carried out with cells at passages 3–5. Once OA SF reached approximately 80% confluence, media usage was switched to DMEM supplemented with 5% EV free FBS (System Biosciences, UK) at 37 °C / 5% CO2 for 24 h before collection of conditioned media (in the presence or absence of IL-1β (2.5ng/ml).

The protocol used for SF isolation was similar to that published in Casnici et al. who confirmed isolated cells were synovial fibroblasts (SF) through expression of the surface marker Thy-1 and negative expression of CD3, CD19, and CD14, as well as morphological confirmation [34]. Images confirming SF morphology and vimentin staining can be seen in Supplementary Fig. 1. Briefly, cells grown on sterile cover slips were washed with PBS and fixed with 4% paraformaldehyde (PFA) for 15 min before being permeabilised with PBS/0.2% Triton X for 10 min, both at room temperature (RT). Cells were blocked with PBS/3% goat serum (blocking buffer) for 45 min at RT before incubation with 1:500 dilution (as per manufacturer’s instructions) of vimentin primary antibody (Ab92547, Abcam) overnight at 4^o^C. Cells were then washed in blocking buffer before incubation with goat anti-rabbit secondary antibody (ab150077, Abcam) for 45 min at room temperature (RT) in the dark. The cells were washed and incubated with the ActinRed™ 555 ReadyProbes™ Reagent (R37112, Thermofisher Scientific) for 30 min at RT in the dark. Finally, cells were washed and mounted onto slides using Dapi ProLong Diamond Antifade Mounting medium (P36971, LifeTechnologies). Images were taken under appropriate channels on the Olympus IX71 Fluorescent Microscope.

For primary articular chondrocyte isolation, cartilage slices were washed in phosphate buffered saline (PBS) with 2% penicillin/streptomycin and 0.5% amphotericin B solution (Sigma-Aldrich, UK). Tissue was cut into small pieces and incubated with sterile 1 mg/ml hyaluronidase in PBS (Sigma-Aldrich) on an orbital shaker for 15 min at 37^o^C at 110 rpm; 2.5 mg/ml trypsin in PBS (Sigma Aldrich) for 30 min at 37^o^C and 1 mg/ml collagenase in complete DMEM (Sigma-Aldrich) for 15–20 h at 37^o^C. Cells were passed through a 70 μm polypropylene with nylon mesh cell strainer (Thermo Fisher Scientific, UK), centrifuged at 1200 rpm for 6 min before resuspension. Articular chondrocytes were cultured in complete DMEM/10% FCS/0.5% amphotericin at 37 °C/5% CO_2_. Experiments with primary chondrocytes were carried out at passage 1 to 2.

### sEV isolation

For sEV preparation, conditioned media from OA SF primary cultures (passage 3–5) were utilised with experimental replicates of *n* = 6. SF were cultured to confluence in T75 flasks. Conditioned media was subsequently harvested and sEV isolated using an adaptation of an established methodology [35]. Briefly, conditioned media was initially centrifuged for 5 min at 5,000 x g, followed by 15,000 × g for 20 min, with the supernatant being retained after each spin and the pellet of dead cells and debris discarded. The collected supernatant was placed into 15 ml ultracentrifugation tubes with Noryl cap assemblies (Beckman Coulter; UK; catalogue numbers 355651 and 355604 respectively) before being centrifuged for 60 min at 100,000 × g at 4^o^C (Beckman Coulter Optima LE-80 K ultracentrifuge; Ti70.1 Beckman Coulter fixed angle rotor) with supernatant discarded, and the sEV pellet retained. The pellet was re-suspended in 1 ml of sterile PBS before further centrifugation for 60 min at 100,000 × g at 4^o^C. The sEV pellet was then re-suspended in either sterile PBS (100 µl for in vitro experiments) or Qiagen lysis buffer (100 µl for RNA preparation) as per assay requirement. In parallel experiments, confluent fibroblast cultures were stimulated with IL-1β (2.5ng/ml) for 24 h, supernatants harvested and used for isolation of sEV as described above.

### Characterisation of isolated sEV

sEV were characterised using scanning and transmission electron microscopy (SEM and TEM respectively), and nanoparticle tracking analysis (NTA). SEM and TEM analysis of isolated sEV were undertaken using previously established methodologies [35, 36]. For all imaging, the JOEL IT100 or JOEL6400 SEM microscopes were used between 6 and 10 kV.

For NTA, sEV preparations were diluted in sterile PBS and injected via a ‘flow through syringe’ into the viewing chamber of the NS300 (Malvern Instrumentation) machine. 100 nm size calibrated standards of CD63-coupled, highly uniform polystyrene spheres, packaged as an aqueous suspension (Malvern Analytical) were used as controls. NTA 3.2 software was used to track the motion of each particle from frame to frame, to calculate particle size and concentration.

### Labelling and uptake of sEV preparations

Isolated sEV preparations were labelled as per manufacturer’s instructions with Exo-Red (stains RNA) or Exo-Green (stains protein), both of which were available as an sEV cargo fluorescent labelling kit (Exo-GlowTM Exosome Labelling Kit, Systems Bioscience, Cambridge Bioscience UK). 1.5 × 10^8^ labelled sEV were subsequently added to primary chondrocytes (1 × 10^5^) cultured on Nunc Lab-Tek II chamber slide systems (Thermofisher, UK) and incubated for 4 h in DMEM supplemented with 5% EV free FBS (System Biosciences, UK). Uptake was monitored using fluorescence microscopy (Olympus IX71 Fluorescent Microscope).

### Viability assay

OA chondrocytes cultured on glass chamber slides (1 × 10^5^ cells) were co-cultured with 2 × 10^10^ sEV for 72 h in in DMEM supplemented with 5% EV free FBS (System Biosciences, UK), after which time cells were washed and treated as per the LIVE/DEAD Viability/Cytotoxicity Kit protocol (Molecular Probes Invitrogen Detection Technologies, UK). Briefly, cells were treated with a combined mixture of 2 µM calcein AM and 4 µM EthD-1 solution, diluted in PBS and imaged under a fluorescent microscope. Cells were counted manually using an average of 4 images per chamber and dead cells normalised to total average cell count.

### qPCR analysis of chondrocyte gene expression

Chondrocytes were seeded at 1 × 10^6^ in 24 well plates in DMEM supplemented with 5% EV free FBS (System Biosciences, UK) and co-cultured with or without 2 × 10^10^ sEV for 6 h. RNA was extracted from chondrocyte cells using Qiagen RNAeasy kit (Qiagen, UK), before undergoing DNase treatment. Synthesis of cDNA took place using Superscript IV First-Strand Synthesis System kit (Invitrogen, UK). Quantitative polymerase chain reaction assay (qPCR) was subsequently used to look at changes in chondrocyte gene expression using the cDNA template, gene specific primers (Thermo Fisher Custom Primers, Invitrogen, UK – see Table 1) and SYBR green chemistry. qPCR was run as per MIQE guidelines [37], on the StepOne qPCR machine (Applied Biosystems StepOnePlus Real-Time PCR System; StepOne Software v2.1) under three steps per cycle with a total of 40 cycles: enzyme activation (2 min at 95 ^o^C); denaturation (10 s at 95 ^o^C) and data collection (60 s at 60 ^o^C). Analysis of qPCR was undertaken by quantification of gene expression which was calculated relative to an average of two normalisation genes; GAPDH and ACTB (initially identified as suitable housekeeping genes using the human geNorm Kit, Primer Design, UK) and normalised to unstimulated controls [38]. Primer sequences for all genes (Thermo Fisher Custom Primers, Invitrogen, UK) are shown in Table 1.

**Table 1:** Primer sequences

Gene (Human)	Forward Primer	Reverse Primer
*GAPDH*	5’ CGCTCTCTGCTCCTCCTGTT 3’	5’ CCATGGTGTCTGAGCGATGT 3’
*ACTB*	5’ CACCATTGGCAATGAGCGGTTC 3’	5’ AGGTCTTTGCGGATGTCCACGT 3’
*MMP13*	5’ AAGGAGCATGGCGACTTCT 3’	5’ TGGCCCAGGAGGAAAAGC 3’
*ACAN*	5’ AGGCAGCGTGATCCTTACC 3’	5’ GGCCTCTCCAGTCTCATTCTC 3’
*COL2A1*	5’ CGTCCAGATGACCTTCCTACG 3’	5’ TGAGCAGGGCCTTCTTGAG 3’
*SOX9*	5’ TGGGCAAGCTCTGGAGACTTC 3’	5’ ATCCGGGTGGTCCTTCTTGTG 3’
*F2RL1*	ATGCGAAGTCTCAGCCTGGCG 3′	5′ GAGAGGAGGTCGGCCAAGGCC 3′
*MMP1*	5′ GGGAGATCATCGGGACAACTC 3′	5′ GGGCCTGGTTGAAAAGCAT 3′

Forward and reverse primer sequences for genes of interest. *GAPDH* = glyceraldehyde 3-phosphate dehydrogenase gene; *ACTB* = β actin gene; *MMP13* = matrix metalloproteinase 13 gene; *ACAN* = aggrecan gene; *COL2A1* = collagen type II genes; *SOX9* = SYR-Box Transcription Factor 9 gene; *F2RL1* = proteinase activated receptor 2 (PAR2) gene; *MMP1* = matrix metalloproteinase 1 gene

### Enzyme linked immunosorbent assay (ELISA)

Impact of sEV on chondrocyte protein secretion was evaluated using commercially available ELISA kits for matrix metalloprotein (MMP)-3, interleukin (IL)-6, 8, 10 and tumour necrosis factor (TNF)-α (Invitrogen, UK). ELISA protocol was as per manufacturer’s instructions. Chondrocytes were seeded at a concentration of 1 × 10^6^ and co-cultured with 2 × 10^10^ sEV for 48 h in 24 well plates, before supernatants were harvested and used for ELISA.

### Dimethylmethylene blue (DMMB) sulphated glycosaminoglycan (sGAG) assay

OA cartilage explants of equal weight per donor (range: 0.47–0.7 g) were incubated with 2 × 10^10^ sEV per 0.5 g tissue over a period of 1 week. Samples of media were taken at day 3 and 7 post sEV exposure to test for the presence of sGAG. sGAG was assayed in harvested supernatants using a spectrophotometric method where 1-9-dimethylmethylene blue (DMMB) is used to create a sGAG-DMMB complex and create a shift in absorbance [39].

### RNA extraction and small (sm)RNA enrichment

RNA was extracted from SF parental cells and their derived sEV. For isolation of RNA from cells, the Qiagen RNAeasy kit (Qiagen, UK) was used as per manufacturer’s protocol. A Total Exosome RNA and Protein Isolation Kit (Invitrogen Life Technologies, UK) was used for RNA isolation from sEVs as per manufacturer’s instructions.

### Library pool preparation

RNA-seq libraries were prepared using the SMARTer^®^ smRNA-Seq Kit for Illumina ^®^ (Catalogue Number 635030; Clontech Laboratories, Inc.). PCR clean-up was performed with NucleoSpin^®^ Gel and PCR Clean-up Gel extraction (Macherey-Nagel). Library quantification was undertaken using the Kapa Library Quantification Kit for lllumina^®^ Platforms (KapaBioSystems; Roche). Working volumes were calculated for each sample to create an equimolar library pool of 5nM.

### RNA-seq and bioinformatics analysis

RNA sequencing was undertaken on the NovaSeq S1 lane as a Low Input Small RNA Library preparation (Edinburgh Genomics, University of Edinburgh, Scotland, UK). The fastQ files were trimmed to 20 bp using cut-adapt and then aligned to the human genome (GRCh38, release 94) using Bowtie, under default settings. Verse was used to count reads (default settings), and differential expression calculated using DESeq2, specifying condition and sample pair as an additional covariate. The data was explored visually using the Searchlight pipeline, under default settings [40–44].

### Statistical analysis

Differences between variables were analysed depending on relevant statistical tests. Multiple variable results were analysed in GraphPad. Regression results and t-tests were undertaken in Excel and GraphPad. Significance levels are indicated as * *p* < 0.05; ** *p* < 0.01 and *** *p* < 0.001. Results are shown as mean ± standard error of mean (SEM).

## Results

### Characterisation of OA SF sEV

For this study, sEV were isolated from OA SF ± IL-1β and subsequently characterised using a variety of techniques.

**Fig. 1:**
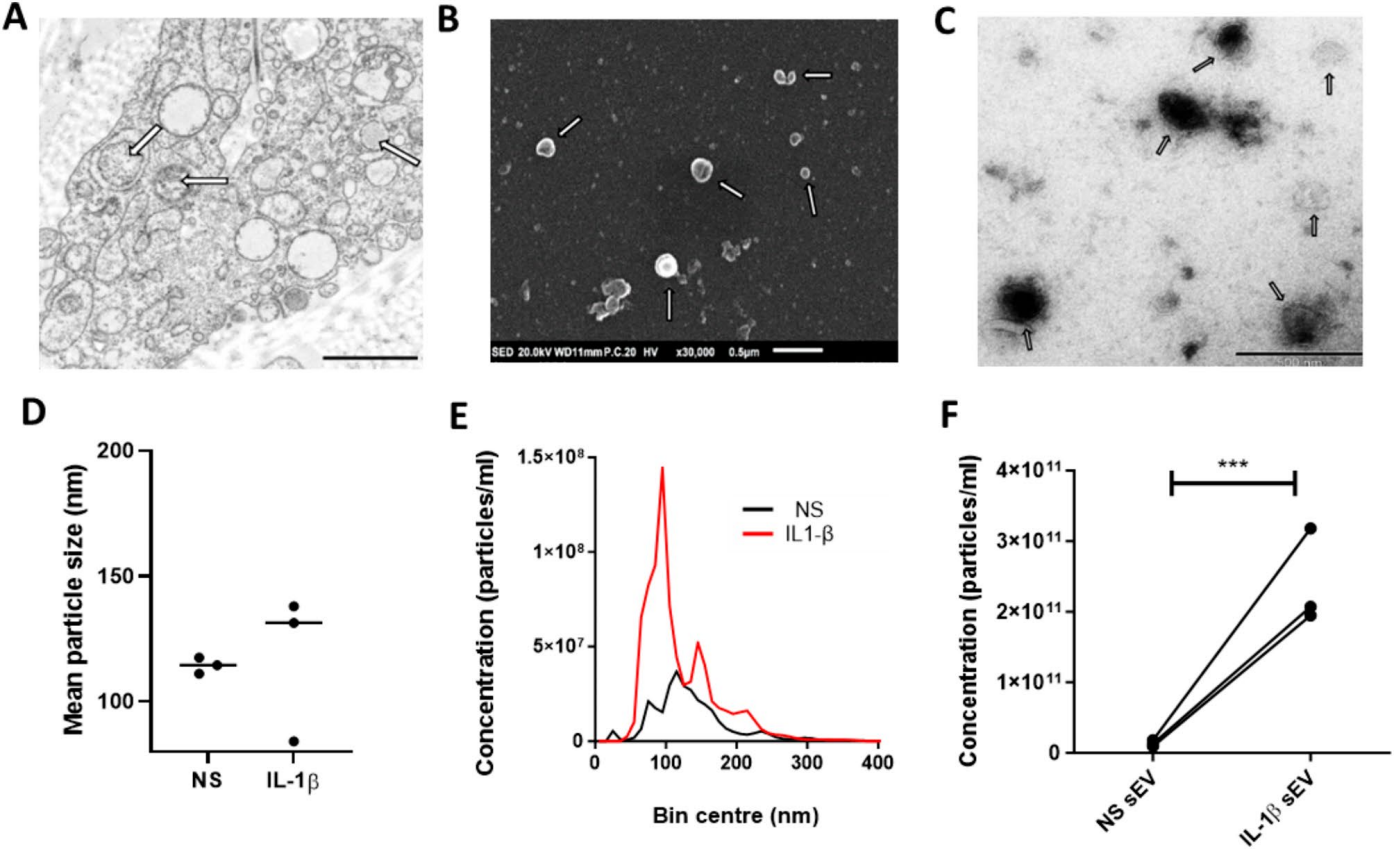
Characterisation of SF sEV by SEM, TEM and NTA analysis. (**A**) Example TEM images of primary SF showing multivesicular bodies packed with sEV (as shown by arrows) at x2,500 magnification. Scale bars 1 μm. (**B**) Example SEM and (**C**) TEM images of SF sEVs preparations; x30,000 - x50,000 magnification. Scale bars 0.5 μm. NTA analysis confirmed the size and concentration of sEV. (**D**) sEV size from 3 separate SF donors ± IL-1β (2.5ng/ml) is shown. (**E**) Example NTA analysis of sEV from one donor. (**F**) Concentration of sEV released from SF ± IL-1β (2.5ng/ml) from *n* = 3 SF donors. NTA – nano tracking analysis. NS = non stimulated. *N* = 3; Significance levels are shown as **p < 0.001, Mann-Whitney test

TEM images of microvesicle bodies (MVBs) in parental OA SF were captured (*n* = 3 donors), with a typical example shown in Fig. 1A. MVBs (> 250 nm) are packaged with sEV during formation and when signalled, move to the cell membrane to release their content. sEV isolated from OA SF conditioned media were confirmed using both SEM and TEM imaging. This was corroborated by the observation of a double membrane (or depression), cup shaped morphology and size range 50–120 nm, Fig. 1B and C. sEV were seen both as isolated and aggregated structures.

NTA analysis found that 80–90% of particles were of the correct sEV size, ranging between 50 and 150 nm with a slight increase in mean particle size with IL-1β stimulation, Fig. 1D. Stimulation of SF with IL-1β significantly increased secreted sEV concentration, Fig. 1E and F (*n* = 3; *p* < 0.001).

### Uptake of sEVs by OA chondrocytes and effect on cell viability

**Fig. 2:**
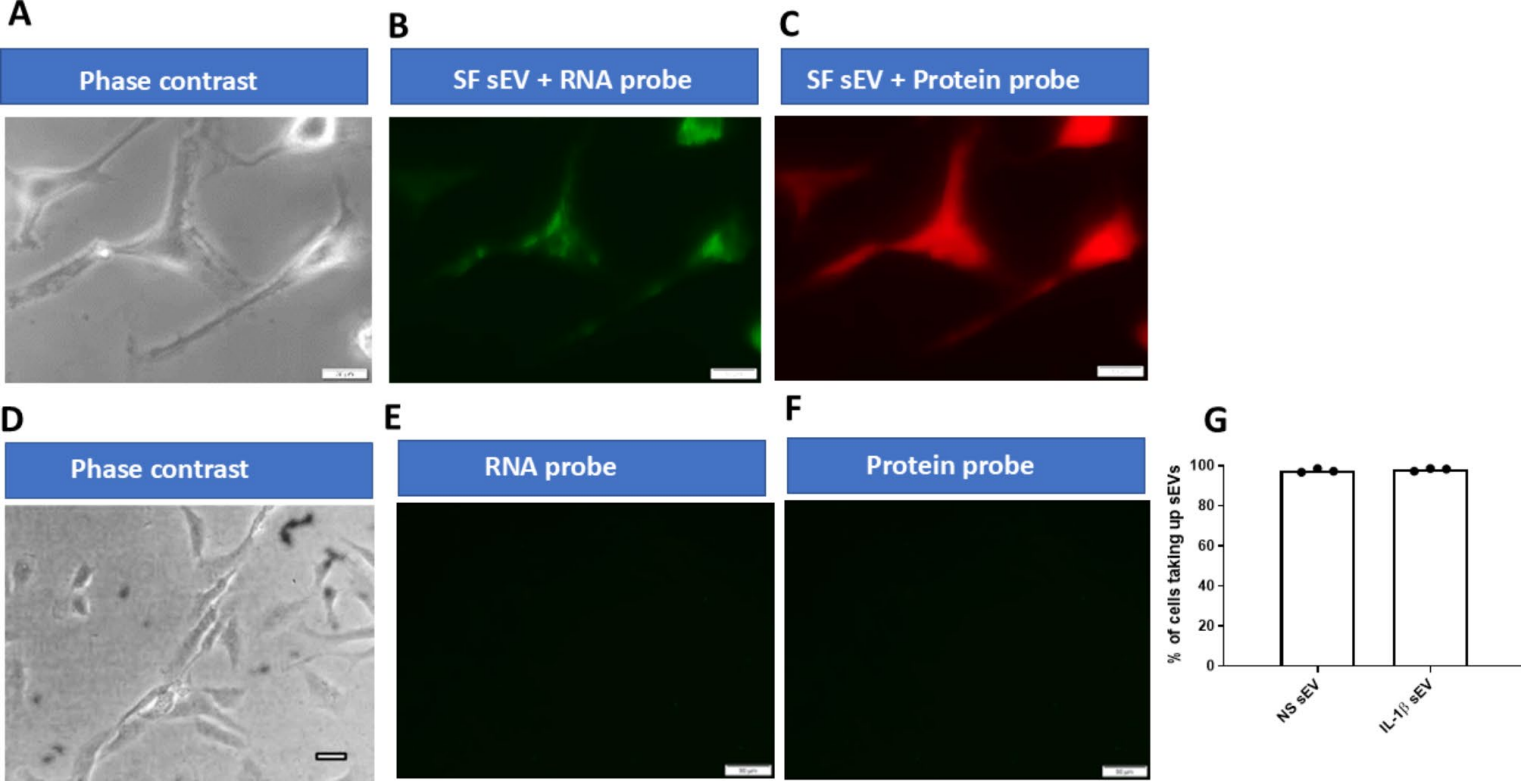
Uptake of OA SF sEV by OA Chondrocytes. (**A**) and (**D**) Example of phase contrast images of synovial fibroblasts (SF). (**B**) Example image of chondrocyte uptake of sEV labelled with RNA (green) or (**C**) protein (red) probe after 4 h incubation. Control SF incubated with (**E**) RNA (green) probe only or (**F**) protein (red) probe. (**G**) There was no difference in the percentage of chondrocytes taking up sEV derived from non-stimulated or IL-1β (2.5 ng/ml) stimulated fibroblasts. Scale Bars 100 μm. NS = non-stimulated; SF = synovial fibroblasts; *n* = 3

**Fig. 3:**
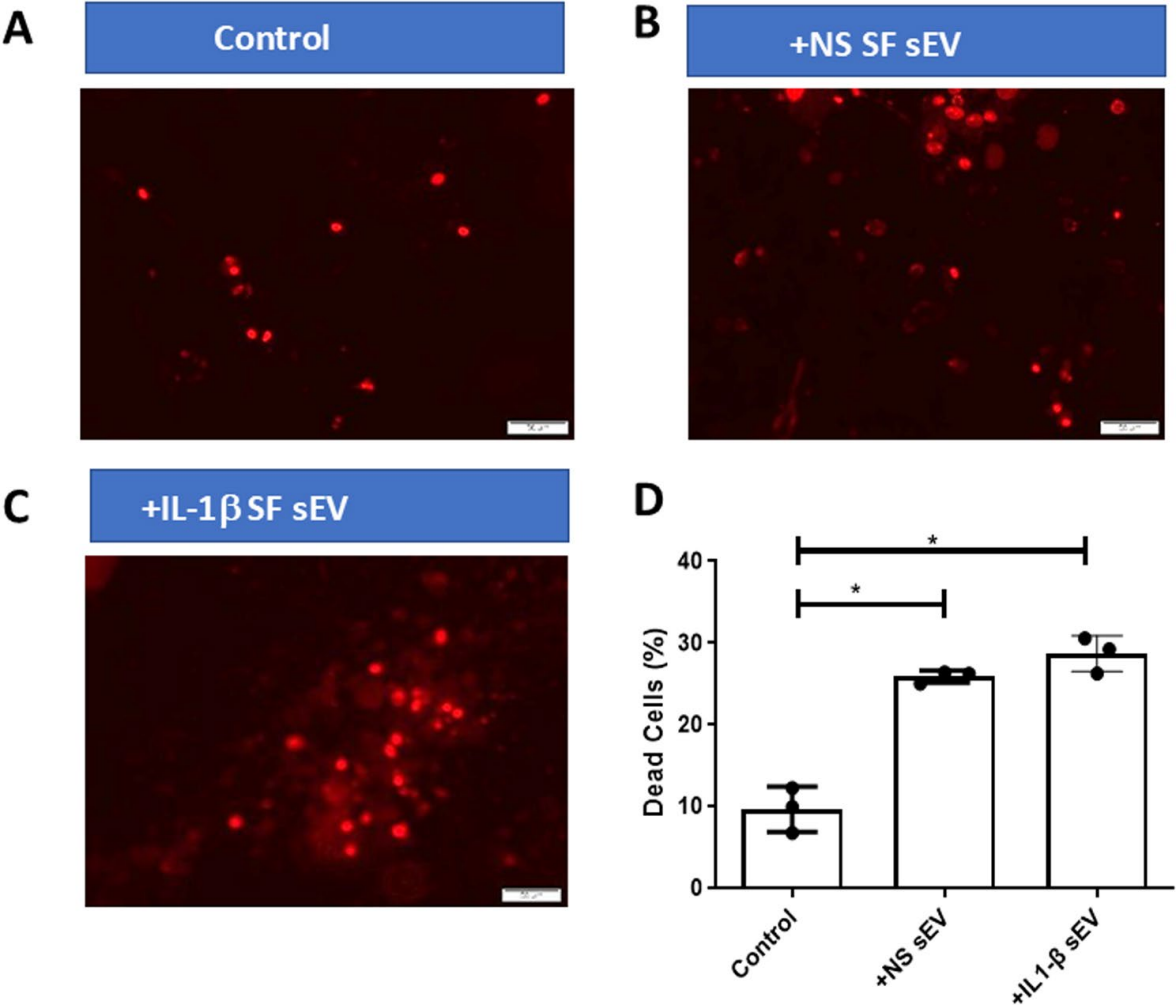
Live/dead staining of OA chondrocytes post exposure to sEV. Phase contrast, dead stain (red) and live stain (green) images. sEV were added in equal concentrations to OA chondrocytes, 72 h after which cells were fixed and stained to determine number of live and dead cells. (**A**) Example OA chondrocyte control dead cell image; (**B**) Example dead cell image after addition of sEV derived from non-stimulated SF; (**C**) Example dead cell image after addition of sEV derived from IL-1β (2.5ng/ml) stimulated SF. (**D**) Summarises the percentage of dead cells seen at 72 h in the presence or absence of sEV. NS = non-stimulated; SF = synovial fibroblasts. *N* = 3; Significance levels are shown as * *p* < 0.05. Comparison of mean values was performed by Mann-Whitney *U*-test

Images confirmed that both non-stimulated and IL-1β OA SF derived sEV were taken up by 80–90% of chondrocytes, Fig. 2A-G. Since chondrocyte death is a known feature of OA cartilage erosion, it was therefore important to established cell viability post uptake of sEV. sEV uptake by chondrocytes after 72 h showed a small although significant increase in the percentage of dead chondrocytes (*n* = 3; *p* < 0.05), Fig. 3A-D. This was observed for both non-stimulated and IL-1β treated SF derived sEV.

### sEV regulation of OA chondrocyte gene expression

**Fig. 4:**
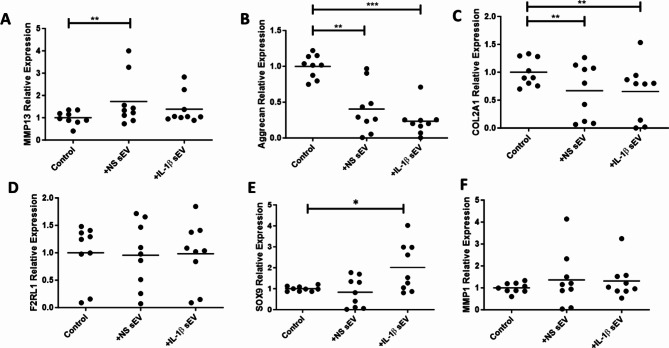
Regulation of OA chondrocytes genes by OA SF sEV. Primary OA chondrocytes were cultured with sEV from OA SF ± IL-1β (2.5 ng/ml) for 6 h and change in gene expression determined by qPCR. Genes for (**A**) *MMP13* (**B**) *Aggrecan* (**C**) *COL2A1* (**D**) *F2RL1 (PAR2)* (**E**) *SOX9* and (**F**) *MMP1*, were normalised to an average of reference genes *GAPDH* and *ACTB* and normalised to unstimulated controls. NS = non-stimulated; SF = synovial fibroblasts. *n* = 9 chondrocyte assays; *n* = 3 sEV preparations. Significance levels are shown as *p < 0.05; **p < 0.01 and ***p < 0.001 compared to a no sEV control (OA chondrocytes only), Wilcoxon test and Friedmans test used

Exposure of OA chondrocytes to SF derived sEVs for 6 h resulted in a significant increase in *MMP13* (*p* < 0.01) gene expression, Fig. 4A, with no significant effect on *MMP1* observed, Fig. 4F. A decrease in *ACAN* and *COL2A1* (*p* < 0.01), Fig. 4B and C, was also observed, with no change in *F2RL1* or *SOX9*, Fig. 4D and E. PAR2, encoded by the *F2RL1* gene, has been identified as a key regulatory check point for OA cartilage erosion [45], however no change in gene expression was noted in chondrocytes post exposure to sEV, Fig. 4D.

Contrastingly, sEV from IL-1 β treated SF did not significantly alter *MMP13* gene expression, Fig. 4A, although they did significantly decrease *ACAN* (*p* < 0.001), and *COL2A1* (*p* < 0.01), while increasing SOX9 (*p* < 0.05), Fig. 4B, C and E respectively. No effect on expression of *F2RL1* or *MMP1* gene expression was observed, Fig. 4D and F, in a similar manner to that seen with sEV from non- stimulated SF.

### sEV regulation of cytokine and proteinase secretion from OA chondrocytes and sGAG from cartilage

**Fig. 5:**
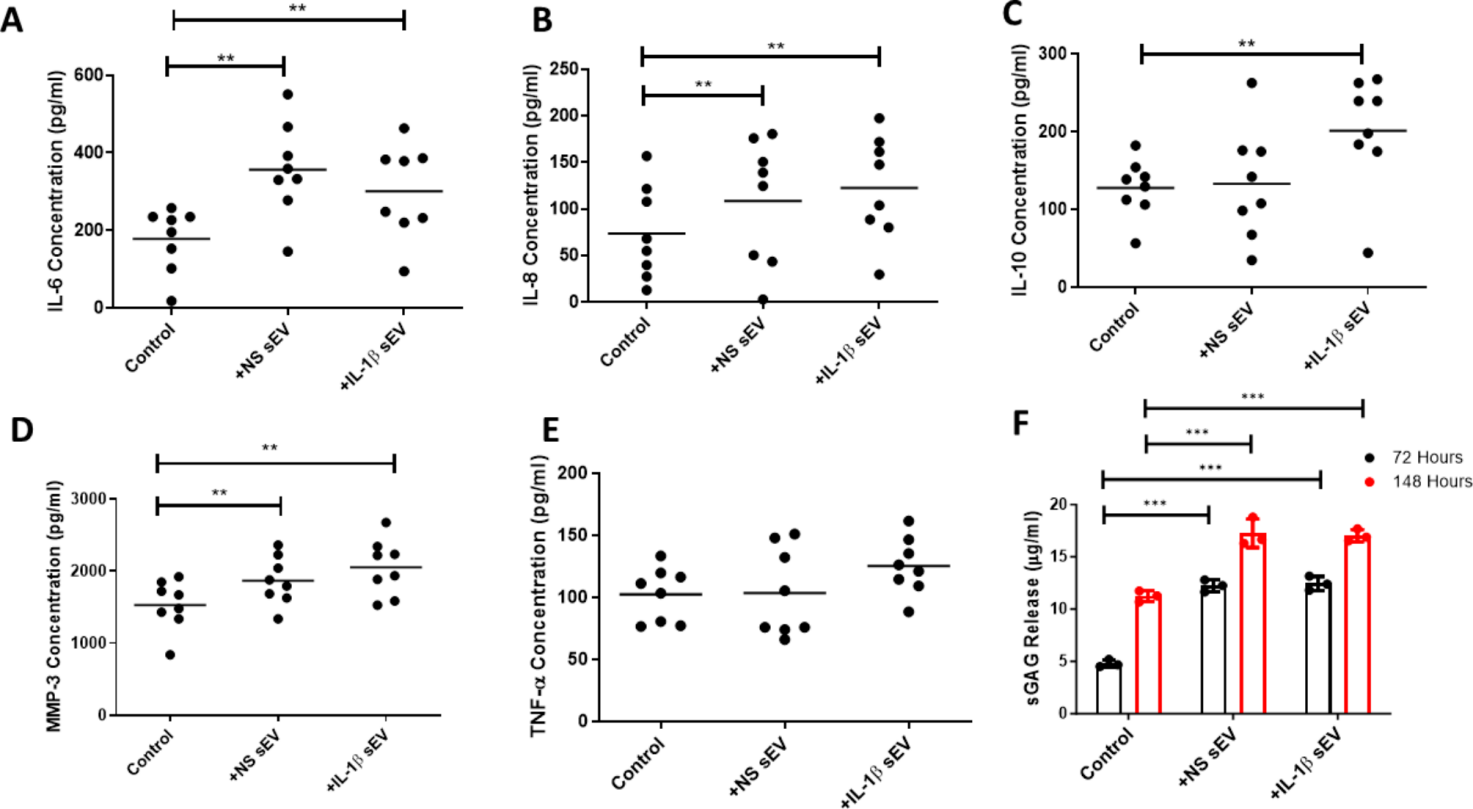
OA SF sEV regulation of inflammatory mediators from OA chondrocytes and modulation of sGAG release from cartilage explants. sEVs derived from SF (± IL-1β) were added to chondrocytes for 24 h. Secreted levels of (**A**) IL-6 (**B**) IL-8 (**C**) IL-10 (**D**) MMP-3 and (**E**) TNF-α are shown. *n* = 8; **p < 0.01 compared to a chondrocyte control with no sEV exposure. (**F**) SF sEV were cultured with cartilage explants and sGAG release evaluated at 72 and 148 h. NS = non-stimulated; SF = synovial fibroblasts *N* = 5–8; Significance levels are shown as * *p* < 0.05 ***p < 0.001, Wilcoxon test and Friedmans test used

sEV derived from non-stimulated (NS) OA SF significantly increased IL-6, IL-8 and MMP-3 (*p* < 0.01) secretion from OA chondrocytes, compared to untreated control cells, Fig. 5A, B and D, with no change in IL-10 or TNF α, Fig. 5C and E respectively.

sEV derived from IL-1 β stimulated OA SF also induced significant increases in IL-6, IL-8 and MMP-3 (*p* < 0.01), Fig. 5A, B and D with no significant effect on TNF α secretion, Fig. 5E. Interestingly, IL-10 was also significantly increased (*p* < 0.01), Fig. 5C, although this was only observed with sEV derived from IL-1β treated SF. Overall, similar cytokine levels were induced in chondrocytes treated with sEV from NS or IL-1β, with no significant difference observed between the two treatments.

sGAG release from OA cartilage significantly increased with exposure to sEV at 72 (*p* < 0.001) and 148 (*p* < 0.001) hours, compared to control cartilage, Fig. 5F. There appeared to be no difference in the ability of sEV derived from NS or IL-1β OA SF to induce sGAG release at 72–148 h.

### miRNA signature of sEVs and parental OA SF

**Fig. 6:**
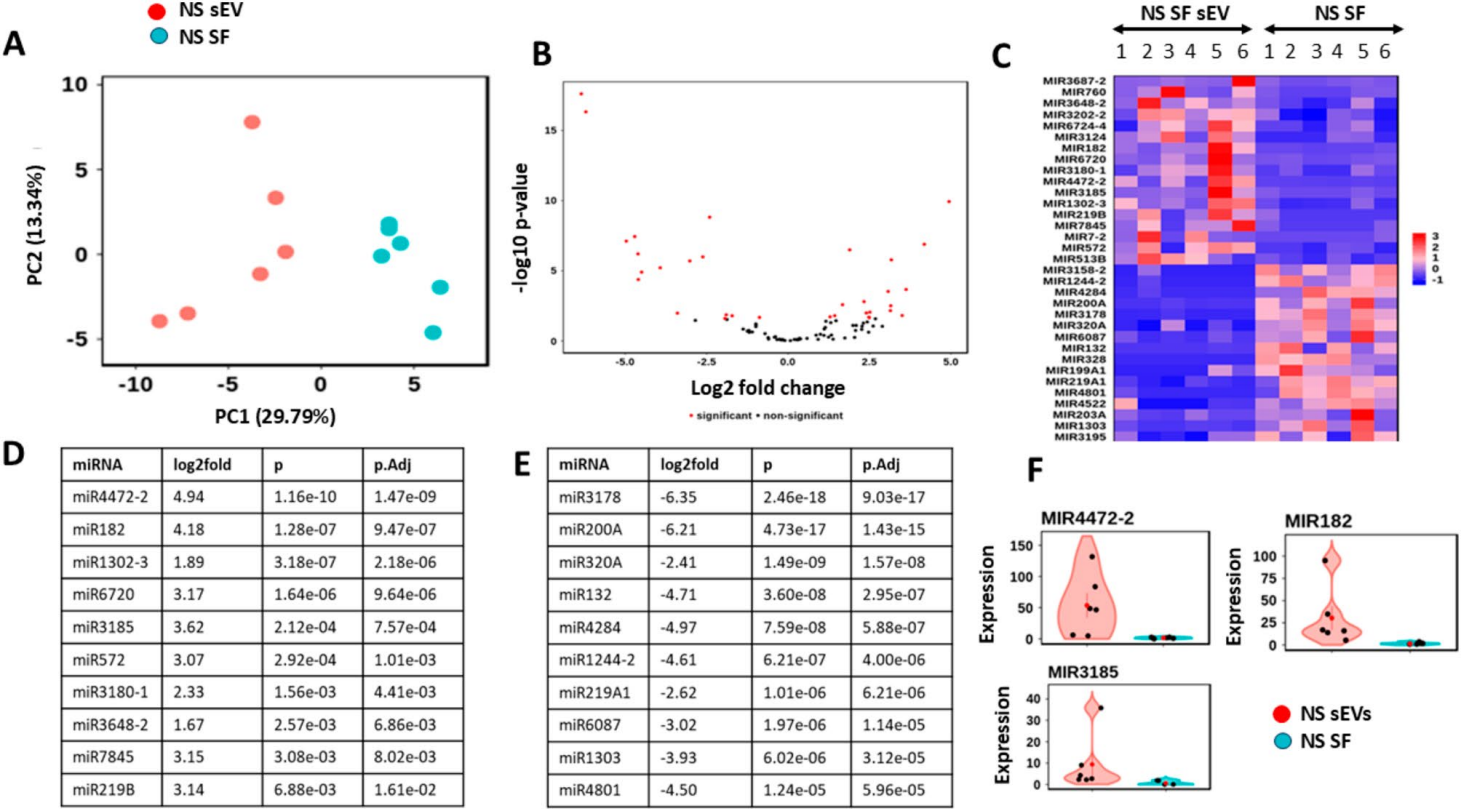
miRNA signatures in OA SF and derived sEV. (**A**) Principal component analysis (PCA) of expression data, showing the first two components. (**B**) Volcano plot, with positive fold change indicating higher expression in NS sEV, compared to parental SF. Significant miRNAs (p.adj < 0.05, absolute log2 fold > 0.0) are shown in red and non − significant miRNAs in black. (**C**) Heat map of differentially expressed miRNA. Samples are on the x axis and genes on the y axis. Colour intensity represents expression level and shows differentiation between groups. Top 10 most highly upregulated (**D**) or down regulated (**E**) miRNA signatures in sEV compared to parental SF, listed by adjusted p values. (**F**) Example violin plots for three of the most highly upregulated sEV miRNA compared with parental SF (*n* = 6). Red dots are sEV and blue dots represent parental SF. NS = non-stimulated; SF = synovial fibroblasts

As there has been much debate as to whether sEV and their parental cells share the same molecular profile, miRNA signatures were determined in both. Utilising 6 OA donors, Principal Component Analysis (PCA) demonstrated a clear separation between miRNA profiles in sEV and their parental OA SF cells (*n* = 6), Fig. 6A. Further investigation found that 17 miRNA were significantly upregulated and 16 significantly downregulated in the sEV when compared to their parental SF cells, Fig. 6B, with the generated heat map in Fig. 6C demonstrating clear differences between the groups. The top ten miRNA signatures significantly upregulated or down regulated in sEV compared to parental SF are shown in Fig. 6D and E respectively. Example violin plots showing differences in expression levels for miR4472-2, miR182 and miR3185 are shown in Fig. 6F, with an overexpression seen in sEV. This data shows for the first time that for OA SF cells, miRNA packaging of secreted sEV is selective and does not mimic the cargo of the parental cell.

### miRNA signature of sEV derived from IL-1β stimulated OA SF

**Fig. 7:**
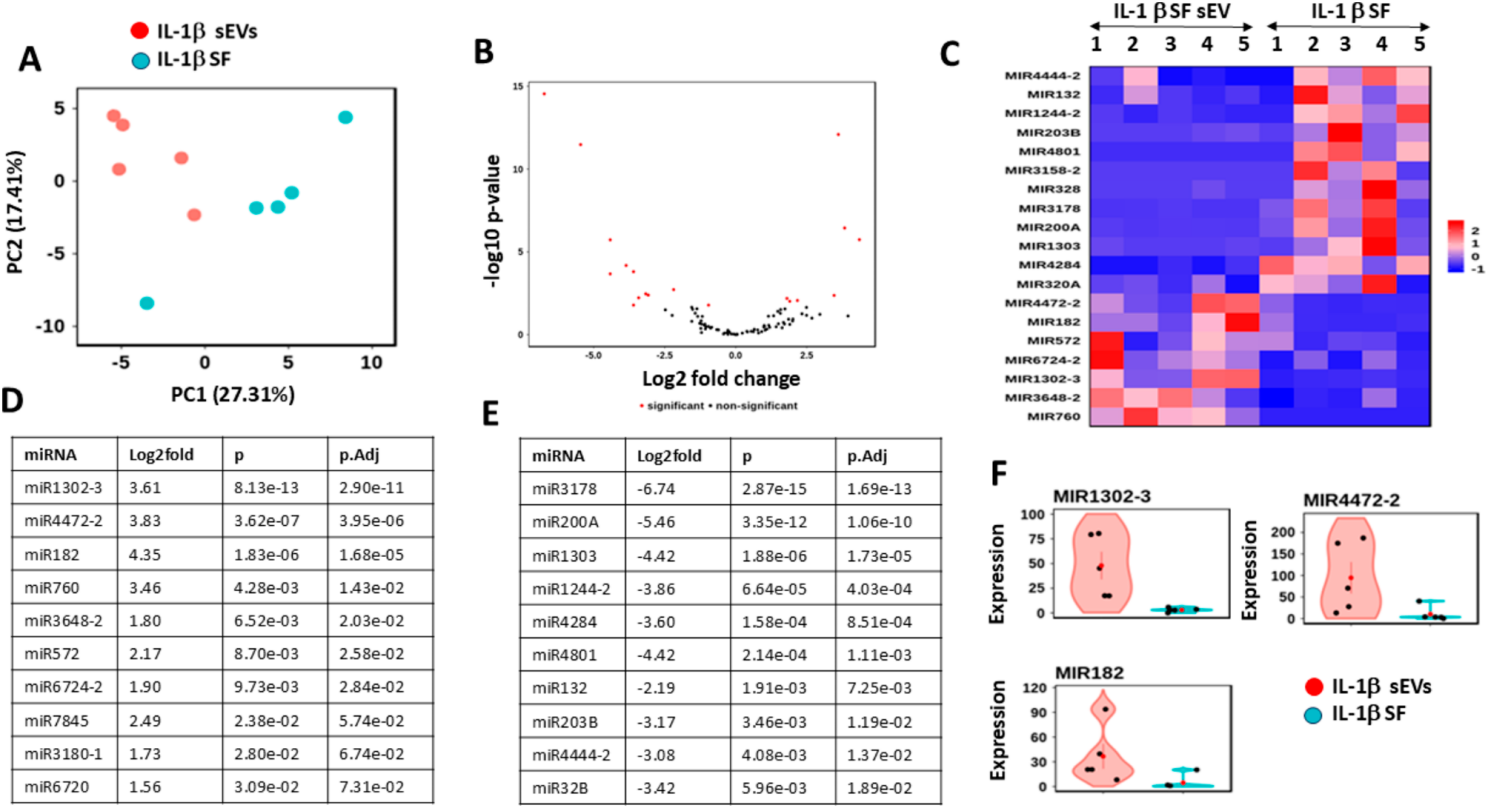
miRNA signatures in IL-1 β stimulated OA SF and derived sEV. (**A**) Principal component analysis (PCA) of expression data, showing the first two components. (**B**) Volcano plot, with positive fold change, indicates higher expression in sEV compared to parental SF stimulated with IL-1β (2.5ng/ml). Significant miRNAs (p.adj < 0.05, absolute log2 fold > 0.0) are shown in red and non − significant miRNAs in black. (**C**) Heat map of differentially expressed miRNA. Samples are on the x axis and genes on the y axis. Colour intensity represents expression level and shows differentiation between groups. Top 10 most highly upregulated (**D**) or down regulated (**E**) miRNA signatures in sEV compared to parental SF, listed by adjusted p values. (**F**) Example violin plots for three of the most highly upregulated sEV miRNA compared with parental SF (*n* = 6). Red dots are sEV and blue dots represent parental SF. SF = synovial fibroblasts

There were distinct differences between sEV from IL-1β stimulated OA SF and their parental cells (*n* = 5) as shown by principal component analysis (PCA), Fig. 7A. Further investigation found that 7 miRNA were upregulated and 12 downregulated in IL-1β sEV compared to parental cells, with the generated heat map showing differences between sEV and parental cells, Fig. 7B and C respectively. The top ten miRNA signatures either enriched or downregulated in the sEVs are shown in Fig. 7D and E, with clear differences seen between sEV and IL-1β treated parental SF, Fig. 7F, as shown in example violin plots.

### IL-1β stimulation of parental OA SF does not significantly alter the sEV miRNA signatures compared to sEV derived from non-stimulated OA SF

**Fig. 8:**
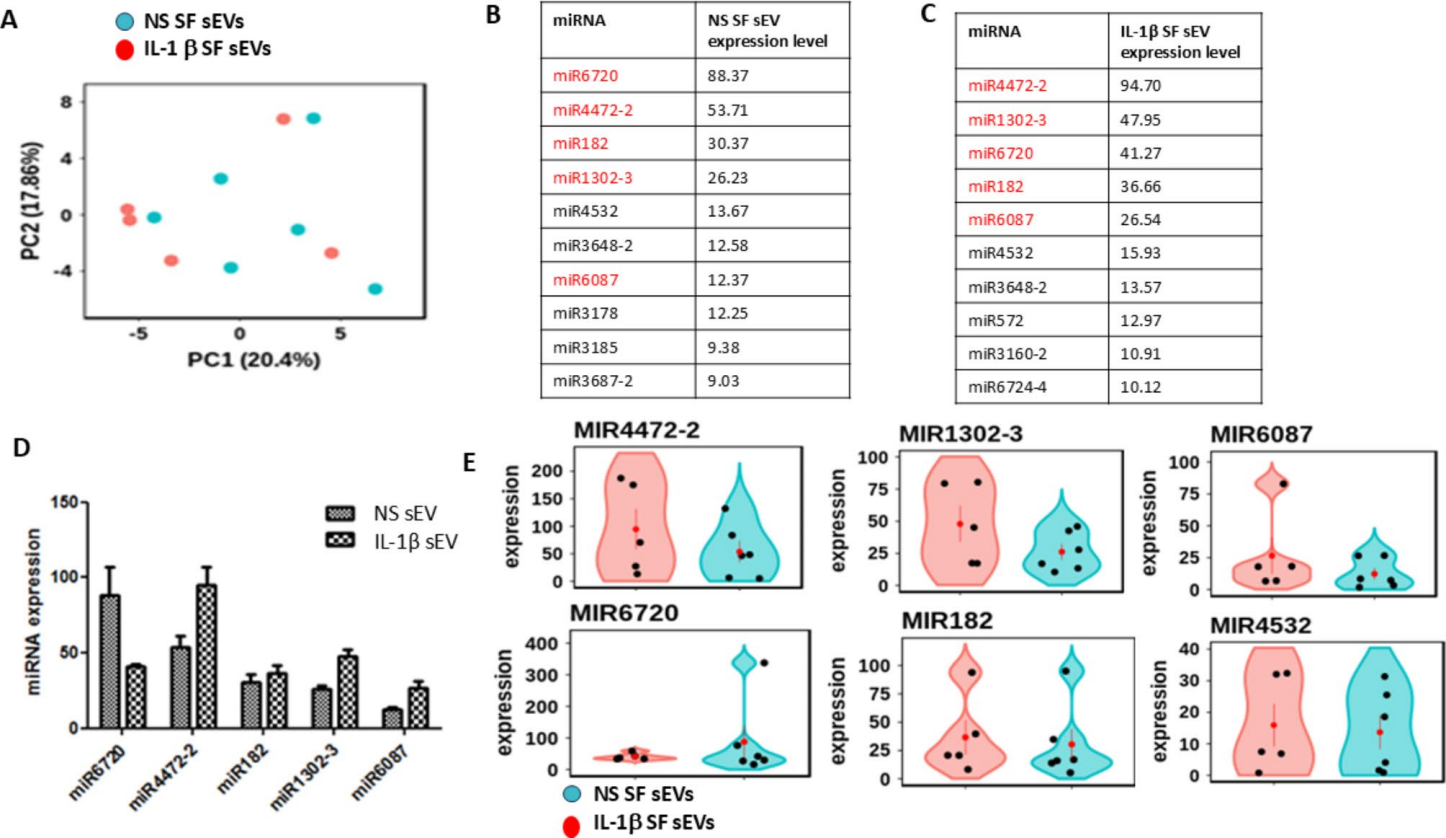
Comparison of miRNA signatures in sEV derived from non-stimulated or IL-1β stimulated OA SF. (**A**) Principal component analysis (PCA) of expression data, showing the first two components. miRNAs are ordered by expression level. (**B**) and (**C**) show the top 10 miRNA enriched in sEV derived from either NS or IL-1β stimulated SF parental cells, with common signatures observed. Significant miRNAs (p.adj < 0.05, absolute log2 fold > 0.0) are shown in red and non − significant miRNAs in black. (**D**) is a graphical representation of the top five miRNA expressed in NS and IL-1β sEVs. Example violin plots for the top 6 miRNA expressed are shown in (**E**). SF (*n* = 6). Blue dots are sEV derived from NS OA SF; red dots are sEV derived from IL-1β stimulated OA SF. NS = non-stimulated; SF = synovial fibroblasts

miRNA signatures were compared for sEV derived from non-stimulated or IL-1β stimulated OA SF. No significant differences were seen between NS and IL-1β OA SF derived sEV miRNA cargo, indicating that the process of selective packaging of sEV is IL-1β independent in OA derived samples. PCA scatterplots showed no differentiation between NS and IL-1β sEV, as shown in Fig. 8A. miRNA profiles between NS and IL-1β sEV were similar as shown in Fig. 8B and C, with miR4472-2, miR1302-3, miR6720, miR182, miR6087 and miR4532 highly enriched, with no significant difference in expression, Fig. 8D. Example violin plots are shown in Fig. 8E.

### TargetScan analysis miR182

Of the 6 most enriched miRNAs identified in the OA SF derived sEV, a search of the literature indicated that miR182 was the most extensively investigated (see Supplementary Fig. 2A). TargetScan analysis resulted in a list of genes, which were predicted to be affected by miR182. A stringent cut off of ‘1000 3P-seq tags + 5’ was put in place. 1000 3P-seq tags + 5, also known as the 3’-UTR profile, are normalised tags that quantify codon usage and hence, give a predictive indication of effect of miRNA on gene regulation. The predicted genes generated by TargetScan were compared with two published studies which had reported differential gene expression in OA chondrocytes [19, 46]. Commonality was seen in 16 genes reported in the study by Chen et al. (2018), summarized in Supplementary Fig. 2B and 13 genes reported by Ji et al. (2019), summarized in Supplementary Fig. 2C.

## Discussion

TEM images showed MVBs of the correct size, packed with sEV sized vesicles which were of similar size and structure to those previously reported [47]. sEV presence was confirmed in line with current ISEV (International Society for Extracellular Vesicles) guidelines [48, 49]. SEM/TEM images of preparations showed vesicles within 50–150 nm with a cupped shaped morphology and double membrane. Interestingly, NTA data showed significant increases in sEV concentration when stimulated with IL-1β, in comparison to basal secretion. This agrees with previous results based on SF derived sEV which showed an increase in concentration with IL-1β inflammation [20], although the cells were derived from normal joint tissues and not OA, a critical difference between the two studies. This increased release of sEV by IL-1B stimulated SF indicates that the sometimes-modest effects seen in our data could be heavily amplified due to the sheer quantity of sEV released in disease state. While previous work with SF has utilised IL-1β in vitro to mimic the inflammatory environment of the OA joint [20], sEV derived from disease relevant tissue and cells is more physiologically relevant and informative. It is also worth noting that sEV prepared from the synovial fluid of OA patients and healthy subjects showed no difference in either concentration or size, indicating that this increase may be specific to SF [21].

Chondrocytes treated with OA SF sEV exhibited sEV uptake and endocytosis, raising the possibility that sEV cargo has a role in regulating chondrocyte fate, ECM degradation and OA pathology. Data presented within this paper shows that addition of OA SF derived sEV elicits a catabolic effect on OA chondrocytes and cartilage explants. sEV addition to OA chondrocyte cultures consistently resulted in an increase in the percentage of dead cells, indicating an influence on cell viability, potentially contributing to the degeneration of cartilage through a catabolic imbalance. This aligns with published results which have shown that articular chondrocytes treated with OA synovial fluid derived sEV have decreased cell survival [50, 51]. We have shown that SF OA derived sEV exacerbates poor chondrocyte ECM maintenance in OA, with addition of OA SF derived sEV causing an increase of sGAG into conditioned media of cartilage explants compared to no sEV controls. Our data agrees with published work showing that mouse femoral head cartilage explants cultured with sEV from non-stimulated fibroblast or IL-1β-stimulated cells increases sGAG and proteoglycan release [20].

The presented changes in chondrocyte gene expression and protein secretion post sEV uptake also aligns with our initial hypothesis. Significant increases in expression of *MMP13*, alongside significant decreases in *COL2A1 and ACAN*, compared to no sEV controls indicate a shift towards catabolic pathways in the chondrocyte. This data aligns with previously published work where sEV isolated from conditioned medium from IL-1β stimulated human non-diseased SF were cultured with articular chondrocytes resulting in upregulated expression of *MMP13* and *ADAMTS-5*, and decreased expression of *COL2A1* and *ACAN* in comparison to sEV collected from non-stimulated SF [20]. Similarly, articular chondrocytes treated with OA synovial fluid derived sEV decreased cell survival and expression of anabolic genes including *COL2A1* and *ACAN* and increased expression of catabolic genes including *IL-6* and *TNF-α* [50, 51]. In the present study, we have demonstrated sEV regulation of secreted inflammatory mediators IL-6, IL-8, MMP-3 and TNF-α from primary OA chondrocytes which would further support our hypothesis. It is also worth noting that we have used a one hit in vitro model in this paper due to limitations in isolation, but it is known physiologically that sEV are consistently released in short bursts and have a much wider impact, indicating that the cumulative effect of diseased derived sEVs could be far greater than what we see in vitro [24, 25]. Previously published work using OA SF sEV has demonstrated their ability to regulate the release of several inflammatory cytokines, chemokines, and metalloproteinases from M1 macrophages [31]. Our current work supports and extends this observation by giving insight into a novel regulatory pathway involving OA SF sEV and chondrocytes with potential relevance for erosive cartilage disease. In our study, little difference is seen between sEV derived for OA SF or OA SF treated with IL-1 β. Interestingly, sEV released from healthy SF treated with IL-1β did show distinct functionalities [20]. Similarly, IL-1β-treated murine articular chondrocytes stimulated catabolic events, whereas sEV isolated from the medium of vehicle-treated chondrocytes inhibited catabolic events and increased messenger RNA levels of *aggrecan* and *type II collagen* in IL-1β-treated chondrocytes [52]. This indicates that the OA derived cells used in this study were unable to respond to IL-1 β due to the damage already undergone by the tissue before surgery or perhaps epigenetic changes. The significance of these findings needs further investigation and while beyond the scope of this study, highlights the importance of using disease relevant cellular models which translate well to physiological conditions.

In our study, 17 miRNAs were increased, and 16 miRNAs decreased in non-stimulated SF derived sEV in comparison to their parental cells, while in sEV derived from IL-1β stimulated SF, 7 miRNA were upregulated and 12 downregulated compared to parental cells. This difference could possibly indicate a mechanism of active and selective packaging by parental cells into sEV. Previous studies have differed considerably in terms of reported sEV content. Indeed, some studies have described protein and RNA content distinct from that of the parental cell, while others have reported sEV populations which carry typical cellular constituents and could potentially be used as biomarkers [27, 53]. Our data contradicts previous reports where sEV RNAs were found to have a similar profile to their parent cell [18, 54, 55] and may reflect our use of disease relevant primary cells. Findings in the OA literature have only recently begun to focus on the role of sEV miRNA cargo. For example, one group found the miRNA content of sEV differed between OA and non-OA groups and between sexes [50]. Furthermore, profiling of miRNA in synovial fluid sEV from OA patients has shown miR-200-c to be increased 2.5-fold compared to synovial fluid from healthy subjects [21].

Importantly, our study has utilised fibroblast and chondrocyte cells derived from OA tissues and may explain some of the differences observed when comparing the results here to other published work. It is possible that the cells used to generate sEV for this paper (all derived from OA synovial membrane fibroblasts), contain an epigenetic stamp from their previous microenvironment which would merit further investigation. The basal inflammatory cytokine release from our primary OA chondrocyte cultures was also found to be higher than that published for studies using normal chondrocytes [56], lending credence to this hypothesis. The impact of OA SF derived sEV on non-OA primary chondrocyte function was not undertaken as we did not have access to normal cartilage and is a limitation of the current study. We have focused on upregulated miRNA as these are the ones that are enriched and would be packaged inside the sEV and carried to recipient cells. miR4472-2, miR1302-3, miR6720, miR182, miR6087 and miR4532 were found to be the most significantly enriched (*p* < 0.001 in comparison to parental cells) and upregulated in comparison to parental cells. This is the first characterisation of these miRNAs in sEV from disease relevant OA SF cells.

Of the signatures identified, miR182 has previously been reported as being upregulated in OA cartilage and chondrocytes [57]. Moreover, miR182 has recently been reported as a key osteoclastogenic regulator in bone homeostasis and diseases [58], with its inhibition resulting in bone protection. TargetScan analysis of miR182 predicted regulation of several genes previously shown in other studies to be differentially expressed in OA chondrocytes [19, 46]. sEV have been shown to be crucial in transport and effect of target cells with breakdown of the sEV membrane with Triton-X stopping phenotypic effects in co-culture [24]. This solidifies the importance of the work presented here which adds to our current understanding of how synovial fibroblasts may regulate cartilage changes in the diseased OA joint, providing insight for future targeted therapy.

While the present study highlights novel miRNA signatures in sEV derived from OA SF, TargetScan analysis here focused on miR182 because of the extensive literature available. Future work looking at the role of the other miRNA identified here is now required. sEV hold enormous promise in determining points of interest for treatments and diagnostic biomarkers. Our study has identified novel miRNA profiles in OA SF derived sEV while giving insight into how the synovial membrane may influence cartilage remodelling. A limitation of this current work centres around the lack of availability of healthy fibroblasts and chondrocytes for comparison, which would provide further evidence to support the importance of this sEV mechanism in OA. Future strategies to enhance the therapeutic potential of sEV could ultimately allow targeted regulation of intercellular communication, improving disease management.

## Conclusions

Using disease relevant tissue and cells, this study demonstrates a role for sEV derived from OA synovial fibroblasts in regulating chondrocyte inflammatory and remodelling responses, highlighting a novel regulatory axis between the synovial membrane and cartilage. For the first time this study shows that sEV derived from OA SF have different miRNA signatures compared to parental cells and that this is not altered with IL-1β treatment. The study has identified novel miRNA signatures in OA SF sEV, including the presence of miRNA182 which is known to have a role in bone homeostasis. Future work looking at the miRNA signatures identified in this study is now required, including their role in OA pathogenesis and potential as disease biomarkers.

## Electronic supplementary material

Below is the link to the electronic supplementary material.

Supplementary Material 1: Morphological characterisation of OA SFs. Example images confirming presence of vimentin on SFs (A) dapi (B) β-actin (C) vimentin and (D) merge. Scale bar 50µm. Magnification x400.

Supplementary Material 2: TargetScan Analysis of miRNA182 and Comparison with Differentially Expressed Chondrocyte Genes. (A) Literature search using PubMed looking at the top 6 miRNA enriched in OA SF sEV found 858 papers on miR182 (yellow), in comparison to the other 5 miRNAs observed, with between 2 and 27 papers found (other colours). TargetScan analysis of miRNA 182 revealed over 1000 target genes. (B) and (C) show genes previously reported to be differentially expressed in OA chondrocytes by Chen (2018) and Ji (2019) respectively [19, 46], and identified as targets of miRNA 182 through TargetScan analysis

## Data Availability

Data sets generated and analysed during the current study are available from the corresponding authors on reasonable request.

## Abbreviations

ACAN: Aggrecan
ADAMTS5: A disintegrin and metalloproteinase with thrombospondin motifs 5
COL2: Collagen type II
DMEM: Dulbecco’s Modified Eagle’s medium
IL: Interleukin
miR: microRNA
MMP: Matrix metalloproteinases
OA: Osteoarthritis
RNA: Ribonucleic acid
RNA-Seq: RNA sequencing
sEV: Small extracellular vesicles
SF: Synovial fibroblast
