# Supplementary figures and images for: Small extracellular vesicles derived from synovial fibroblasts contain distinct miRNA profiles and contribute to chondrocyte damage in osteoarthritis
Source: Arthritis Res Ther. 2024 Sep 28;26:167. doi: 10.1186/s13075-024-03398-3 (PMC11437673; doi:10.1186/s13075-024-03398-3)

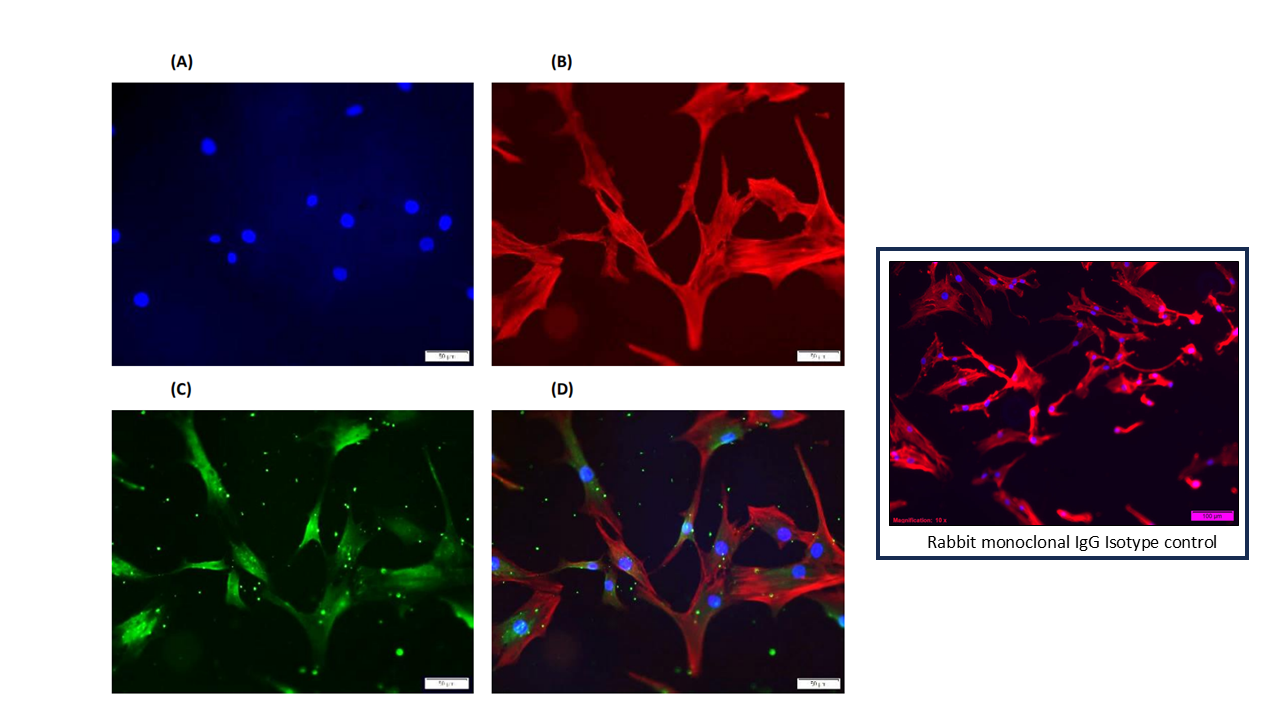

Supplement: Supplementary file 1 — Supplementary Material 1: Morphological characterisation of OA SFs. Example images confirming presence of vimentin on SFs (A) dapi (B) β-actin (C) vimentin and (D) merge. Scale bar 50µm. Magnification x400. [file 13075_2024_3398_MOESM1_ESM.tif]

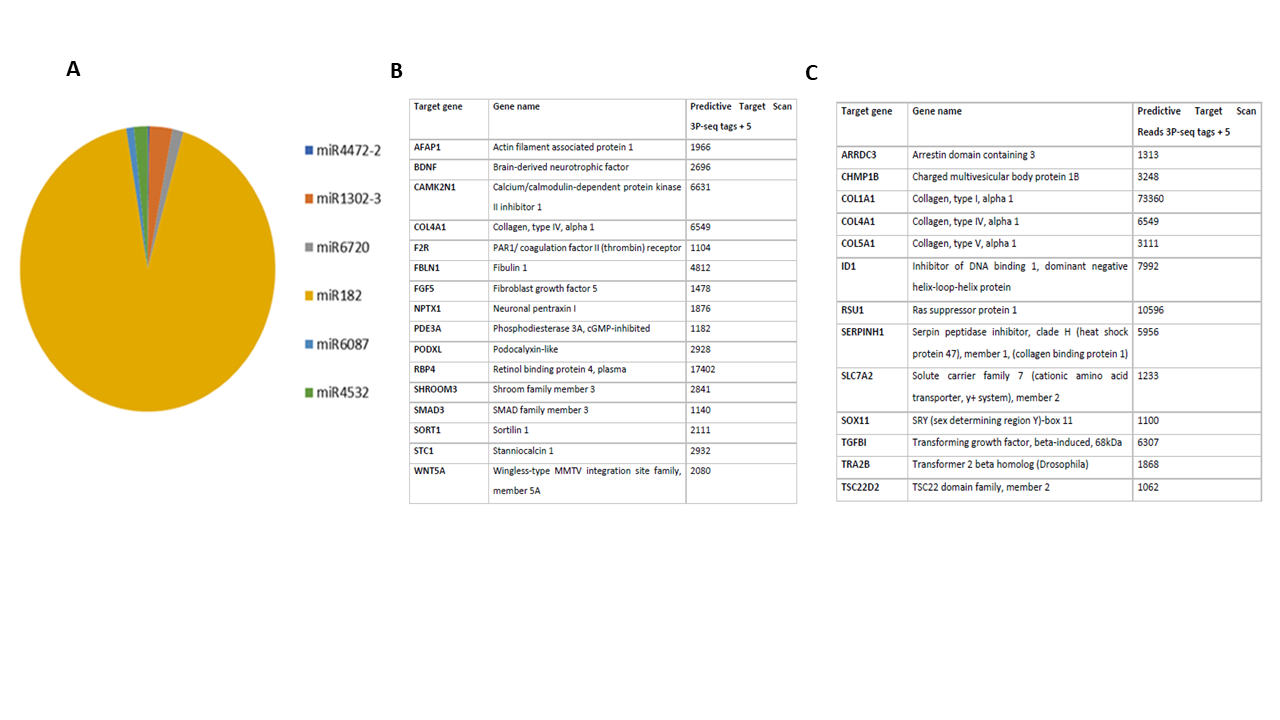

Supplement: Supplementary file 2 — Supplementary Material 2: TargetScan Analysis of miRNA182 and Comparison with Differentially Expressed Chondrocyte Genes. (A) Literature search using PubMed looking at the top 6 miRNA enriched in OA SF sEV found 858 papers on miR182 (yellow), in comparison to the other 5 miRNAs observed, with between 2 and 27 papers found (other colours). TargetScan analysis of miRNA 182 revealed over 1000 target genes. (B) and (C) show genes previously reported to be differentially expressed in OA chondrocytes by Chen (2018) and Ji (2019) respectively [19, 46], and identified as targets of miRNA 182 through TargetScan analysis [file 13075_2024_3398_MOESM2_ESM.tif]
